# Auto‐dentin platelet‐rich fibrin matrix is an alternative biomaterial for different augmentation procedures: A retrospective case series report

**DOI:** 10.1002/cre2.808

**Published:** 2023-11-07

**Authors:** Abdusalam Alrmali, Muhammad H. A. Saleh, John Mazzocco, Jacob M. Zimmer, Tiziano Testori, Hom‐Lay Wang

**Affiliations:** ^1^ Department of Periodontics and Oral Medicine University of Michigan School of Dentistry Ann Arbor Michigan USA; ^2^ Department of Oral Medicine, Oral Pathology, Oral and Maxillofacial Surgery University of Tripoli School of Dentistry Tripoli Libya; ^3^ Section of Implant Dentistry and Oral Rehabilitation IRCCS Orthopedic Institute Galeazzi, Dental Clinic Milan Italy

**Keywords:** autologous dentin graft, bone substitutes, platelet‐rich fibrin, ridge augmentation

## Abstract

**Objectives:**

Autologous dentin grafts derived from extracted teeth have shown promise as bone graft materials for promoting bone regeneration. This retrospective case series aimed to evaluate clinical, radiographic, and histologic outcomes of using autologous dentin matrices in various bone regeneration procedures.

**Materials and Methods:**

This case series included 26 eligible patients and encompassed 4 socket preservation cases, 5 cases of guided tissue regeneration, 5 cases of guided bone regeneration (GBR), 10 cases of sinus augmentation procedures, 2 immediate placement implants, and 2 socket shields. Dentin grafts were prepared from extracted teeth, cleaned, and processed. These grafts were combined with platelet‐rich fibrin (PRF) to create adhesive dentin matrices, then covered with collagen membranes for simultaneous guided bone augmentation cases. Cone beam computed tomography (CBCT) scans were conducted before surgery and 4 months postoperatively to assess ridge dimensions. Histologic evaluation was performed through bone core biopsies for socket preservation cases at the 4‐month mark.

**Results:**

A total of 42 implants were placed in 26 patients, with an average follow‐up of 32 months. Notably, two implant failures occurred following lateral maxillary sinus augmentation. CBCT scans at the 4‐month interval revealed bone coverage over implant platforms in the majority of cases. Histologic analysis from two cases of socket preservation demonstrated dentin granules enveloped by newly formed bone undergoing continuous remodeling. The quantitative histomorphometric assessment revealed a bone area of 42.8 ± 3.56%, a remaining graft area of 19.05 ± 4.58%, and a viable bone of 38.15 ± 7.84%.

**Conclusions:**

The utilization of autologous dentin particles mixed with PRF proved effective as an alternative to conventional bone graft materials in GBR and maxillary sinus augmentation procedures. Larger controlled clinical trials are recommended to further substantiate these findings.

## 1 INTRODUCTION

Different types of graft materials have been described in the literature for use in the treatment of alveolar ridge defects. Current findings show that the use of various grafting materials augmentation procedures results in more favorable outcomes compared to procedures without grafting (Avila‐Ortiz et al., 2019). Autogenous bone is still considered the gold standard material due to its osteogenic potential (Morjaria et al., 2014). However, harvesting autogenous graft material is not indicated in all cases. This is because it presents certain drawbacks such as rapid resorption, limited availability from intraoral sources, and potential issues with patient acceptance (Hallman & Thor, 2008; Nkenke & Stelzle, 2009). To address the limitations of autogenous grafts, alternative materials like allografts, xenografts, and alloplasts have been developed. These grafts not only possess osteogenic qualities similar to autologous grafts but also incorporate osteoinductive and/or osteoconductive mechanisms that enhance their regenerative potential (Khan et al., 2005). Dentin grafts have shown great potential in promoting bone healing for various applications (Pohl et al., 2020). Their osteoinductive potential arises from the presence of bone morphogenetic proteins within the demineralized dentin matrix, which can stimulate new bone formation without causing inflammation (Ferreira et al., 2018). These advantageous attributes, along with the ability to facilitate rapid new bone growth, make dentin grafts a cost‐effective and efficient alternative to traditional graft materials (Andersson, 2010; Hammerle et al., 2008; Murata et al., 2022). The similarities in content and embryonic origin between teeth and bone allow dentin grafts to provide similar benefits to an autogenic cortical bone matrix (Bakhshalian et al., 2013; Nampo et al., 2010). Consequently, dentin grafts are considered autografts, leading to better tissue response compared to grafts derived from other sources (Park et al., 2015). During the healing process, dentin particles undergo ankylosis just like reimplanted avulsed teeth that become ankylosed by bone deposition directly on the cementum and dentin (Bessho et al., 1991). A systematic review of dentin grafts reported histological signs of new bone formation just 2 weeks after grafting, demonstrating the osteoinductive property of the material (Gual‐Vaques et al., 2018).

Dentin graft materials exhibit similar characteristics to conventional bone materials when they are combined with platelet concentrates like platelet‐rich plasma, platelet‐rich fibrin (PRF), and concentrated growth factors. This combination creates a cohesive matrix that not only improves the handling of the graft material but also enhances its stability during procedures (Koga et al., 2016). Moreover, these biological additives have been shown to generate growth factors that play a crucial role in promoting wound healing and the regenerative processes of tissues (Kobayashi et al., 2016). Second‐generation platelet concentrates, that is, leukocyte and PRF, as introduced by Dohan et al. (2006), were also utilized to supplement the particulate dentin graft. The primary objective of this study was to evaluate the clinical, radiographic, and histological outcomes of using autologous tooth matrices for various applications in the fields of periodontics and implant dentistry.

## 2 MATERIALS AND METHODS

### 2.1 Study design

This retrospective study focused on individuals with edentulous ridges located in either the maxilla or mandible. These individuals had impacted or compromised teeth that required extraction, and they were seeking the placement of an implant‐supported prosthesis to address their dental needs.

The study protocol underwent rigorous ethical scrutiny and received approval from the Ethics Committee, specifically for the collection and analysis of data. Surgical procedures were carried out on all patients within a timeframe spanning from 2018 to 2021. These surgeries took place at three private clinics located in Tripoli and Misurata, Libya. Importantly, all surgical interventions were performed by a single, experienced surgeon, namely, A. A.

Patients who participated in this study provided explicit written consent. They were informed that data collected during their routine visits would be included in the study in an anonymous manner for statistical analysis. As an essential aspect of ensuring patient privacy and confidentiality, specific patient files and data were meticulously safeguarded. It is worth highlighting that this analysis adhered to the ethical principles outlined in the World Medical Association Declaration of Helsinki, ensuring that the study was conducted with full regard for the rights and well‐being of the participants.

### 2.2 Study populations and treatment protocol

Twenty‐six patients were included in the study. Twenty patients were nonsmoking adults, over 18 years old. Among them, 18 patients were medically healthy, while 8 patients had well‐controlled diabetes mellitus with no contraindication for ridge augmentation and implant placement. Simultaneous guided bone regeneration (GBR) procedures were performed for 26 implants, lateral wall sinus augmentation for 12 implants, socket shield technique for 2 implants, and immediate implants for 2 implants. The average follow‐up period was 32.03 months. Preoperative and 4‐month postoperative cone beam computed tomography (CBCT) scans were conducted to evaluate the ridge dimensions before and after grafting (Table 1).

**Table 1:** Overall findings of the study, providing a summary of the patient data and outcomes.

Procedure	Socket shield	Guided bone regeneration	Socket preservation	Immediate placement	Closed sinus lifting	Open sinus lifting	Periodontal regeneration
Number of cases	2	5	4	2	8	2	5
Condition of the teeth	Nondecayed erupted third molars	Nondecayed and periodontally involved teeth	Restored teeth but not root canal treated	Periodontally involved	Nondecayed	Periodontally involved	Impacted teeth
Number of teeth used for dentin graft	2	20	4	2	4	5	5
No of implants	2	26	/	2	8	4	/
Number of diabetic patients	/	2	/	2	2	2	/
Failed implants	/	/	/	/	/	2	/

### 2.3 General protocol

All of the patients underwent phase one hygienic periodontal therapy, which included supra‐ and subgingival debriding of the compromised teeth to decrease the level of presurgical inflammation and improve patient compliance. Systemic antibiotic treatment with oral amoxicillin was initiated 1 h before surgery and continued every 12 h for 5 days.

### 2.4 Surgical protocol and graft preparation

#### 2.4.1 Preparation of autologous dentin graft

The process began by thoroughly cleaning and preparing the extracted teeth using a specialized tooth milling device, following the manufacturer's guidelines. Our protocol included several steps, including tooth fragmentation, disinfection, and partial demineralization, all conducted before utilizing the teeth as graft material for GBR or sinus augmentation procedures.

**Figure 1:**
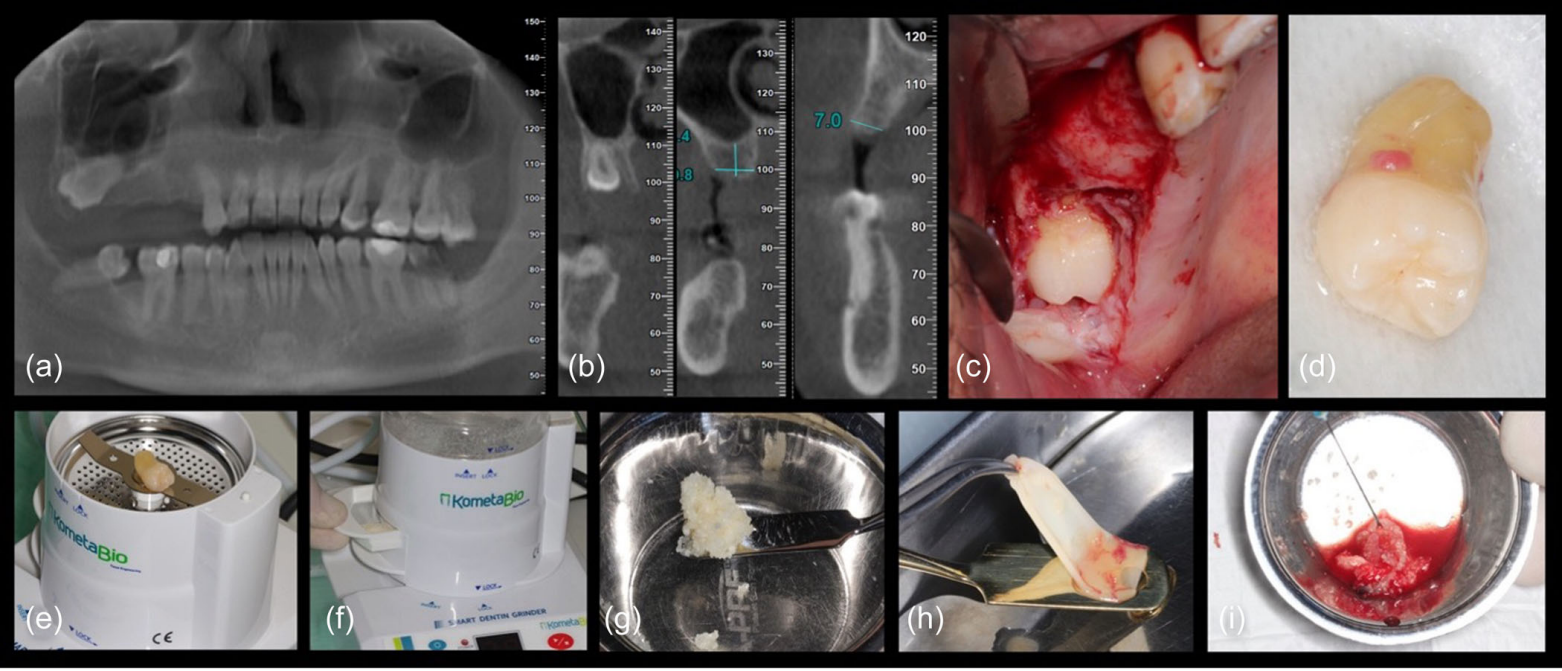
Case of vertical sinus lifting performed simultaneously with implant placement (a, b) present formatted panoramic and coronal views of cone‐beam computed tomography, illustrating the impacted upper third molar and the dimensions of the ridge width and height, (c) displays a full‐thickness flap, revealing the impacted molar, (d) shows the extracted molar, while (e–g) illustrate the grinding process, and (h, i) demonstrate the platelet‐rich fibrin (PRF) and autogenous dentin combined to form a PRF matrix.

To create the autologous dentin graft, any restorative materials present on the extracted tooth were removed using a high‐speed handpiece. However, it's important to note that due to the absence of published studies providing guidance on the complete removal of filling materials, sterilizing liquids, or cement before the grinding process, such teeth were not included in this study's surgical protocols. Furthermore, both enamel and cementum were meticulously eliminated from the extracted tooth before further processing. Afterward, the tooth was dried using sterile gauze and then placed into a specialized dentin grinding device known as the Smart Dentin Grinder (KometaBio) for additional processing (Figure 1).

The dentin grinding process generates graft particles within the size range of 250–1200 µm. To ensure the removal of bacteria and any residual organic material, a high‐pH cleanser known as Dentin Cleanser (KometaBio), containing sodium hydroxide in 20% ethanol, is introduced to these particles. Following a 5‐min exposure to the cleanser, any excess moisture is effectively removed by using sterile gauze. Optionally, ethylenediaminetetraacetic acid can be employed to partially demineralize the particles, a step that exposes more collagen and promotes enhanced site activation during the early stages of the healing process. Subsequently, a dentin washer (Dentin Washer; KometaBio) containing phosphate‐buffered saline is added to the mixture, allowing it to sit for a brief period. After this second soaking, any residual liquid is meticulously eliminated using sterile gauze (Figure 1). The graft material is now fully prepared for use.

To improve its handling characteristics, the autologous dentin graft particles are combined with PRF to form a gelatinous mass before placement (Kobayashi et al., 2016; Koga et al., 2016) (Figure 1). The choice of graft type and particle size depends on the specific application and the required duration for the graft to remain in place. Variations in the grinding and sorting protocols during tooth processing may result in the production of either smaller or larger particles, impacting the overall rate of graft resorption and the integration of new bone at the treated site. Smaller particles may be preferable for addressing small defects like infrabony periodontal pockets or immediate implant‐socket gaps, facilitating quicker new bone formation. Conversely, cases involving large sockets or the absence of bony walls may necessitate a higher proportion of larger particles to maintain the material's scaffolding properties for an extended duration.

**Figure 2:**
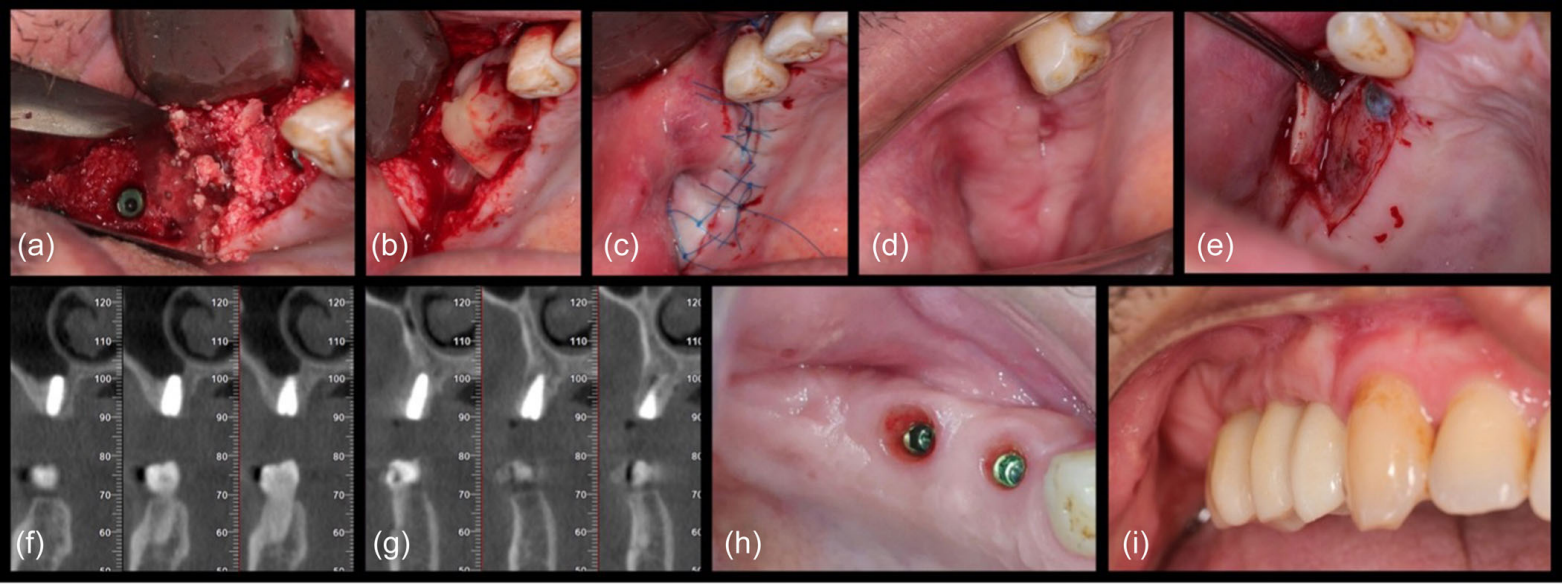
The following stages of the procedure: (a, b) The application of the autogenous dentin platelet‐rich fibrin matrix before and after implant placement. (c) The closure of the flap. (d) The healing process is 2 weeks postoperatively. (e) An apically displaced flap was performed during the second stage. (f, g) Cone beam computed tomography images were taken at 4 months. (h) Situation 4 weeks after placing the healing abutment. (i) 12‐month follow‐up.

In instances where implants were inserted concurrently with augmentation procedures, the dentin grafts were mixed with a PRF solution. This mixture created a sticky dentin matrix that not only facilitated clinical manipulation but also enhanced the stability of the graft material. In all cases, collagen membranes were employed to cover the graft, with an additional PRF membrane applied for additional protection (Figure 2).

#### 2.4.2 PRF preparation protocol

Before commencing the surgical procedures, blood samples were obtained from each patient using a 21‐G vein needle. Approximately 6–8 collection tubes, each containing 9 mL of blood, were filled during this process. Significantly, these plain tubes were used without the addition of anticoagulants. Clinicians were offered recommendations to improve the optimization of PRF clots/membranes by gaining a deeper understanding of PRF tubes (Miron et al., 2021).

Following the blood collection, it was promptly subjected to centrifugation using an Intra‐Spin EBA 200 centrifuge device from the IntraLock System. The centrifugation process applied a force of around 400*g* (or 2700 rpm) for a duration of 12 min. To create l‐PRF (Leukocyte‐Platelet Rich Fibrin) membranes, an Intraspin centrifugation device with specific settings was used. These settings included a rotor angulation of 33°, a 50 mm radius at the clot, and an 80 mm radius at the maximum (Miron et al., 2019).

For this process, 9‐mL glass‐coated plastic tubes provided by Intra‐Lock were utilized. After centrifugation, the tubes were positioned vertically in a rack to allow for the blood to clot for approximately 15–20 min. Following this incubation period, the clot, now transformed into PRF, was gently removed from the collection tubes and placed into a PRF box. A slight amount of pressure was applied to the PRF clot within the PRF‐Box to shape it into a membrane, still using equipment from the Intra‐Lock System (Miron et al., 2018).

**Figure 3:**
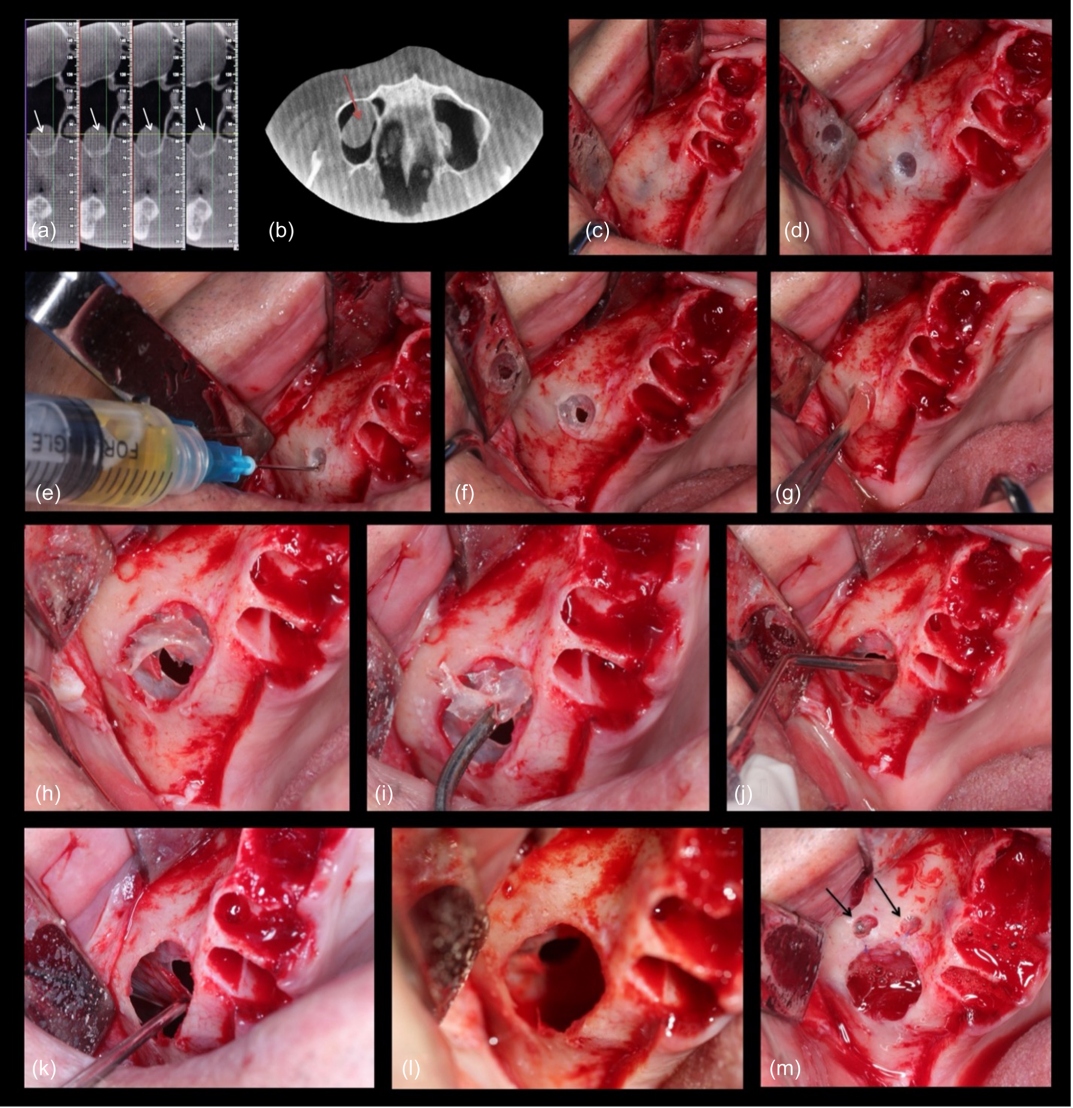
Presents a case involving the surgical removal of a maxillary sinus mucocele carried out simultaneously with sinus lifting (a, b) display coronal and axial views of the cone‐beam computed tomography, highlighting the mucocele (indicated by white and red arrows), (c) a full‐thickness flap revealing the shadow of the sinus, (d) a small opening made to expose the mucocele, followed by the use of a disposable syringe to aspirate the cystic fluid (e). The sinus opening is then enlarged, and intentional perforation is performed (f). The cyst lining is held using small forceps (g), and the opening is further enlarged (h) to allow for the removal of the bone shell (i). Sinus lifting is initiated using various sinus elevators (j, k), leading to complete sinus lifting (l). Finally, two small holes are created to suture the perforated membrane with suture material (m).

**Figure 4:**
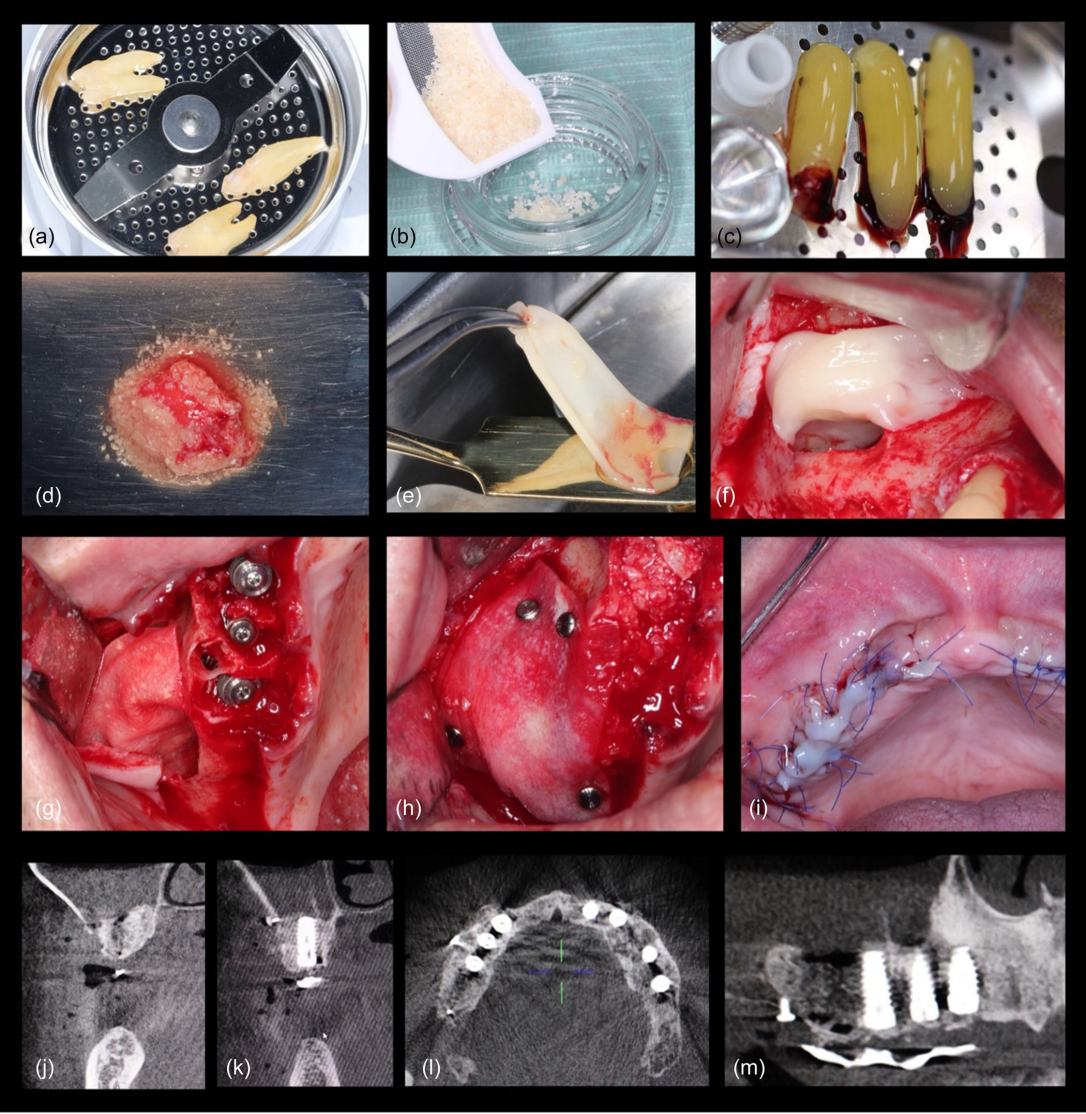
Showcases the following steps of the procedure: (a) Extracted teeth are placed in the grinding machine. (b) The resulting dentin graft with particle sizes ranging from 250 to 1000 μm. (c) Platelet‐rich fibrin (PRF) was prepared. (d) The dentin graft is mixed with the liquid form of PRF. (e, f) PRF membrane was utilized to cover the sinus membrane before placing the dentin graft. (g, h) Collagen membrane employed for intra and extra‐sinus applications. (i) Flap closure. (j–m) Various cone beam computed tomography views captured after sinus augmentation.

To create a sticky dentin matrix graft, the PRF exudate was mixed with the dentin graft. Subsequently, after the graft was placed, the flap was released by scoring the periosteum to achieve primary closure. The flap was then sutured using 5‐0 polypropylene sutures from Ethicon. Postoperative instructions and medication were provided to the patients, and thorough follow‐up care was administered. Visual representations of these procedures can be seen in Figures 3 and 4.

#### 2.4.3 Histological examination

In cases involving socket preservation (two cases), we collected histological samples using trephine burs (specifically, 08.910.03 from Brasseler USA) with a 2 mm diameter. This collection was performed at the time of implant placement, with an average delay of approximately 4 months. The harvested bone samples were carefully preserved in a 10% neutral buffered formalin solution. Subsequently, these samples were sent to the Department of Pathology at the Faculty of Medicine, Misurata University in Libya for thorough histological analysis.

To prepare the bone core biopsies for analysis, we followed a precise protocol. Initially, the biopsies were fixed immediately in a 4% paraformaldehyde solution for a period of 24 h. Subsequently, a decalcification process was carried out using Osteodec (Bio Optica 05‐MO3005) for a total of 6 days, maintaining them at room temperature. After completing this decalcification process, the samples underwent multiple rinses.

**Figure 5:**
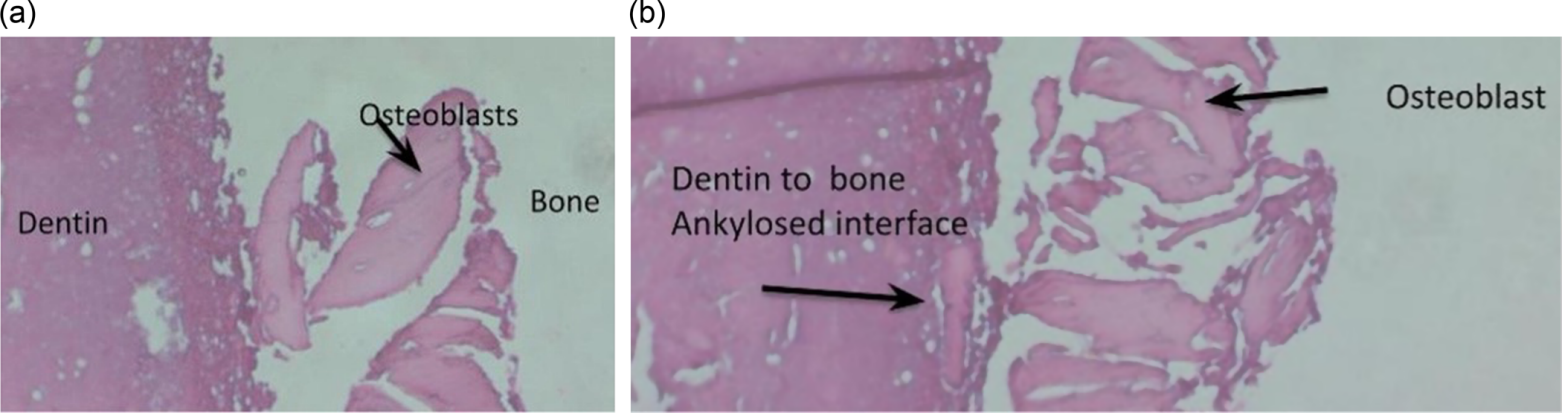
Bone core obtained from an augmented socket using the autogenous dentin platelet‐rich fibrin matrix (a), new bone formation is observed, with cellular osseous tissue in direct contact with the dentin graft particles and (b) a higher magnification view of the marked area, revealing osteoblast‐enriched osseous tissue fused with the dentin graft particles. This fusion indicates replacement resorption of the grafted material. The images were obtained through hematoxylin and eosin staining at ×4 and ×20 magnifications, respectively.

Following the rinsing steps, we subjected the samples to a dehydration process involving a sequence of ethanol solutions. Subsequently, they were cleared in xylene and embedded in paraffin. Microtome sections with a thickness of 3 μm were meticulously prepared to facilitate histological and immunohistochemistry analysis. For qualitative assessment, the morphology of both dental and bone tissues was evaluated on hematoxylin and eosin‐stained slides. The slides were specifically sourced from Thermo Fischer Scientific. To further analyze the histological images obtained, we digitized them through a digital camera. We then employed image analysis software to measure the bone volume, the volume of the remaining dentin graft, and the volume of vital bone. This quantification was conducted using the ImageJ program (version 1.8.0_72; National Institute of Health) (Figure 5).

## 3 RESULTS

Forty‐two implants were placed in 26 patients (17 females and 9 males), with a mean age of 57.2 years. The mean follow‐up for the dental implants was 32.03 months. A total of 42 implants were inserted (19 cases in maxilla, 7 cases in mandible). The case series encompassed 4 socket preservation cases, 5 cases of guided tissue regeneration, 5 cases of GBR, 10 cases of sinus augmentation procedures, 2 immediate placement implants, and 2 socket shields. Table 2 presents the total volume of dentin graft material that can be generated per tooth.

**Table 2:** Total volume of dentin graft material that can be generated per tooth.

Condition of extracted teeth	Total number of teeth	Percentage from the total teeth number	Amount of dentin graft (cc)
Periodontally involved	25	59.5%	1.5–2 cc/tooth
Erupted third molars	2	4.7%	2–2.5 cc/tooth
Impacted third molars	5	11.9%	2–2.5 cc/tooth
Badly decayed	4	9.5%	0.75–1.5 cc/tooth
Root canal treated	None	/	/

Abbreviations: cc, cubic centimeter; %, percentage.

When considering minor complication rates, the temporary exposure of the augmented site was observed in 1.04% of cases. Mild infection of the recipient site was noted in 0.52% of sites. These minor complications did not affect the treatment prognosis, and the implants were inserted as planned. With the exception of two implants that failed following lateral maxillary sinus augmentation in two well‐controlled diabetic patients, the remaining inserted implants were accepted as satisfactory for survival. No major or permanent complications were reported in terms of graft loss or permanent graft exposure. The satisfactory survival rate at the 32.03‐month follow‐up was 95.2% (Table 3).

**Table 3:** Reported complications during the study.

Complications	2	Total number of implants	42	Healthy patient	18
Temporary graft exposure	2	Implant failure	2	Well‐controlled diabetes	8
Mild infection	2			Smoking patients	2 patient, less than 10 cigarette/day
Permanent graft exposure	None	Implant survival rate	95.2%	Immunocompromised patient	None
Infection with graft loss	None	After 3 years follow up	95.2%	HIV patients	None

Abbreviation: HIV, human immunodeficiency virus

After 4 months, the second stage surgery was done for all cases. CBCT volumetric analysis revealed bone covering the head of the implant in most cases. The histological analysis demonstrated dentin granules surrounded by newly formed bone with ongoing remodeling of the graft biopsy after 4 months. Quantitative histomorphometric analysis showed the proportion of the mean of total bone volume, residual graft, and soft tissue area documented in Table 4.

**Table 4:** Results of the quantitative histomorphometric analysis, which measured the proportions of mean total bone volume, residual graft, and soft tissue area.

Total bone volume, %	42.8 ± 3.56
Residual graft, %	19.05 ± 4.58
Vital bone, %	38.15 ± 7.84

## 4 DISCUSSION

Over the years, dentin graft has gained recognition for its unique ability to stimulate bone growth. It can serve as an alternative graft material for various bone augmentation procedures, ridge preservation, and sinus augmentation (Pohl et al., 2020). Dentin and autologous matrix cortical bones have structural similarities which explain their behavioral similarity. Kim et al. (2014) conducted a comparison study with traditional grafting materials showing these physicochemical similarities between dentin and cortical bone. Autologous extracted teeth can be utilized as block grafts for lateral ridge augmentation or processed into particulate dentin and have been shown to undergo gradual resorption replacement similar to autogenous bone (Calvo‐Guirado et al., 2018; Del Canto‐Diaz et al., 2019; Schwarz et al., 2016). Pohl et al. (2016) used CBCT radiography to demonstrate the effectiveness of dentin graft material in preserving alveolar ridge dimensions after tooth extraction. The easy chair‐side preparation protocol and cost‐effectiveness make this material a viable option for filling a wide spectrum of bone defects, as they provide sufficient volume (Calvo‐Guirado et al., 2019).

A comparative investigation that involved samples collected at various time points (specifically, 4, 5, and 6 months after grafting) disclosed a consistent and gradual increase in the amount of bone present, alongside a decline in the content of residual dentin graft (Schwartz et al., 2000). These results are in alignment with the findings of Mazor et al. (2019), who reported a relative bone content of 63% after 7 months, accompanied by minimal remnants of mineralized dentin particles. Additionally, experimental data has demonstrated that mineralized dentin particles exhibit porosity of up to 44.48%. This porosity serves to enhance blood supply and facilitates the gradual resorption of the grafted material, ultimately supporting the healing process and enabling the replacement resorption necessary for the formation of lamellar bone (Miron et al., 2018).

Notably, in the current study, histological analysis including two socket preservation cases revealed the presence of dentin granules surrounded by newly formed bone, which suggests an ongoing process of graft remodeling. The residual dentin graft accounted for approximately 19.05% with a standard deviation of 4.58%, and there were no indications of inflammation or fibrous encapsulation in the histological findings.

PRF plays a crucial role in expediting site healing by providing concentrated quantities of blood derivatives that enhance the activity of macrophages and growth factors, as supported by existing research. Proponents of PRF underscore the notably high concentration of growth factors it contains, with a particular focus on platelet‐derived growth factors (PDGF). Several studies have addressed the impact of silica/silicone coatings on plastic or glass tubes and their potential toxicity to human cells when implanted. Clinicians should consider the role of centrifugation tubes and chemical additives in PRF clot outcomes. As per a previous technical note, its purpose was to warn clinicians about potential risks associated with using tubes containing chemical additives in PRF production. These additives could interfere with or harm tissue regeneration at implantation sites (Miron et al., 2021). PDGF is of paramount importance as it regulates the migration, proliferation, and survival of mesenchymal cells, which are vital for tissue repair and regeneration. Additionally, PRF includes transforming growth factor (TGF)‐β1, a growth factor released by autologous bone. TGF‐β1 plays a significant role in stimulating the synthesis of type 1 collagen, fibronectin, and vascular endothelial growth factor (VEGF). VEGF is a potent factor that promotes tissue angiogenesis, the formation of new blood vessels, which is crucial for ensuring an adequate blood supply during the healing process (Andrade et al., 2020; van Orten et al., 2022). Dentin, on the other hand, serves as a scaffold for tissue regeneration and provides long‐term maintenance at the site. It is important to note that a systematic review reported inconclusive findings regarding the effect of PRF on bone regeneration (Miron et al., 2017). In line with other recent studies, the histologic evaluation of our study supports the potential of the dentin matrix as a valuable contributor to defect fill and optimal healing (Cervera‐Maillo et al., 2021; Minetti et al., 2022). In addition, the combination of autogenous dentin particles and PRF in this study resulted in successful ridge augmentation, even without primary soft tissue closure. Histological analysis confirmed the biodegradable resorption and replacement of the grafted particles, as evidenced by comparing the pre‐extraction and post‐grafting situations at 1 year (Artzi et al., 2022).

In our study, 2 out of 10 sinus lifting patients were experienced with early postoperative infections within the first 2 weeks after surgery. Both patients underwent open sinus lifting, one of whom was a current smoker and who had mucocele removal from the maxillary sinus during the same visit as the sinus lifting procedure. Both patients were treated with vigorous mouth rinses and systemic antibiotics. It is worth noting that this finding is not consistent with the study by Pohl et al. (2016), and it is possible that only roots of completely impacted teeth were used in our study to minimize the potential risk of contamination. During the 32‐month follow‐up period, the average peri‐implant bone resorption was 0.2 mm, with no cases exceeding 2 mm. Two implants were lost during the second stage, and both cases were from patients who had experienced infection within the first 2 weeks postoperatively. Despite these complications, the overall clinical and radiographic findings indicated successful outcomes with high patient satisfaction.

The limitations of this study include small sample size, a relatively short follow‐up period, the absence of a control group, potential bias in patient selection, possible operator variability, lack of randomization and blinding, and the omission of certain external factors. These limitations should be considered when interpreting the results and suggest the need for further research to address these constraints and enhance the generalizability and validity of the findings.

## 5 CONCLUSIONS

A matrix composed of auto‐dentin particles mixed with PRF has been successfully utilized as a substitute for bone substitutes in different augmentation and regenerative procedures.

Further research with a larger sample size and controlled trials is both necessary and ideal to validate the findings of this case series.

## AUTHOR CONTRIBUTIONS

**Abdusalam Alrmali**: Conceptualization; formal analysis; investigation; data curation; project administration. **Abdusalam Alrmali** and **Muhammad H. A. Saleh**: Methodology and resources. **Abdusalam Alrmali** and **Hom‐Lay Wang**: Validation. **Abdusalam Alrmali**, **John Mazzocco**, **Jacob M. Zimmer**, and **Muhammad H. A. Saleh**: Writing—original draft preparation. **Tiziano Testori** and **Hom‐Lay Wang**: Writing—review and editing; **Muhammad H. A. Saleh**: Visualization. **Tiziano Testori** and **Hom‐Lay Wang**: Supervision.

## CONFLICT OF INTEREST STATEMENT

The authors declare no conflict of interest.

## Data Availability

The data that support the findings of this study are available from the corresponding author upon reasonable request.
